# Complete plastome sequence of *Jacaranda mimosifolia* D. Don (Bignoniaceae): a beautiful landscaping tree species

**DOI:** 10.1080/23802359.2019.1692702

**Published:** 2019-11-20

**Authors:** Kun-Kun Zhao, Ren-Li Chen, Jian-Hua Wang, Zhi-Xin Zhu, Guo-Zheng Shi, Shui-Xing Luo, Hua-Feng Wang

**Affiliations:** a The Experimental Station of the Research Institute of Tropical Forestry, Chinese Academy of Forestry, Jianfeng Town, PR China;; b Key Laboratory of Tropical Biological Resources of Ministry of Education, School of Life and Pharmaceutical Sciences, Hainan University, Haikou, PR China

**Keywords:** *Jacaranda mimosifolia*, Illumina sequencing, complete chloroplast, phylogenetic analysis, Bignoniaceae

## Abstract

*Jacaranda mimosifolia* is a deciduous arbor with blue flowers native to Brazil, Bolivia, and Argentina in South America. After introduction from South America, it was widely cultivated as a garden ornamental plant in South China. The complete chloroplast (cp) genome sequence of this ornamental species is reported in this study, based on high-throughput sequencing (Illumina). The complete cp genome is 153,514 bp in length, containing a pair of inverted repeat regions (IRs) of 25,408 bp, a large single-copy (LSC) region of 84,755 bp, and a small single-copy (SSC) region of 17,943 bp. The cp genome contains 130 genes, consisting of 85 protein-coding genes, 37 tRNA genes, and 8 rRNA genes. The overall A/T content in the cp genome of *J. mimosifolia* is 61.70%. The phylogenetic analyses indicate that there is a close relationship between *J. mimosifolia* and *Tecomaria capensis*. The complete cp sequence of *J. mimosifolia* will provide a useful resource for the development and utilization of this species as well as for the phylogenetic studies in Bignoniaceae.

*Jacaranda mimosifolia* is a deciduous arbor belonging to the Jacaranda of Bignoniaceae, native to Brazil, Bolivia, and Argentina in South America. Its leaves are graceful and resemble *Albizia julibrissin*, the fruit is peculiar and resembles tortoise shell and the flowers are blue and bell-shaped. It is a very good ornamental plant for green, widely cultivated in Guangdong, Guangxi, Yunnan, and Hainan of China (Yang and Li 2011). Here, we report and characterize the complete chloroplast of *J. mimosifolia* (GenBank accession number: MN450152). This is the first report of a complete chloroplast for the *J. mimosifolia*.

We sampled a healthy individual of *J. mimosifolia* in this study from Baishamen Park, Haikou City (N20.07°, E110.33°). A voucher specimen (Wang et al., B263) was deposited in the Herbarium of the Institute of Tropical Agriculture and Forestry (HUTB), Hainan University, Haikou, China. We employed the modified CTAB method (Doyle and Doyle 1987) to extract the total genomic DNA of *J. mimosifolia* from silica gel-dried leaves.

Whole-genome short-gun sequencing was performed on the Illumina Hiseq 2500 platform (Illumina, San Diego, CA), with the 150 bp paired-end sequencing method. We carried out quality control of sequenced genomic data and cleaning up unqualified sequences (Patel and Jain 2012). Finally, about 6 GB of clean data is obtained. The *J. mimosifolia* was assembled by MITObim version 1.8 (omicX, France, Hahn et al. 2013). All genes were annotated by Geneious R version 8.0.2 (Biomatters Ltd., Auckland, New Zealand). The chloroplast genome of *Erythranthe lutea* (NC_030212.1) (Vallejo-Marín et al. 2016) was used as the reference for assembling and annotation. The annotation was corrected with DOGMA (Wyman et al. 2004).

The chloroplast genome of *J. mimosifolia* was a circular molecule of 153,514 bp with the typical quadripartite structure of angiosperms, containing two inverted repeats (IRs) of 25,408 bp, a large single-copy (LSC) region of 84,755 bp, a small single-copy (SSC) region of 17,943 bp. The chloroplast genome contains 130 genes, including 85 protein-coding genes, 37 tRNA genes, and 8 rRNA genes. The overall A/T content in the chloroplast genome of *J. mimosifolia* is 61.70%, which the corresponding value of the LSC, SSC, and IR region were 63.70%, 67.10%, and 56.60%, respectively.

A maximum-likelihood (ML) phylogenetic tree of the nine published complete chloroplast genomes of Bignoniaceae (plus *J. mimosifolia*) was built with RAxML (Stamatakis 2006), using *Utricularia reniformis*, *Pinguicula ehlersiae*, *Salvia japonica*, and *Sesamum indicum* as outgroups (Figure 1). The phylogenetic analysis indicated that all members of Bignoniaceae were clustered with a high bootstrap support (BS) value and there was a close relationship between *J. mimosifolia* and *Tecomaria capensis*. In this study, we report the characterization of the complete chloroplast genome of *J. mimosifolia* for the first time, which may provide a useful resource for development and utilization of *J. mimosifolia*, and also for phylogenetic studies of Bignoniaceae.

**Figure 1.**
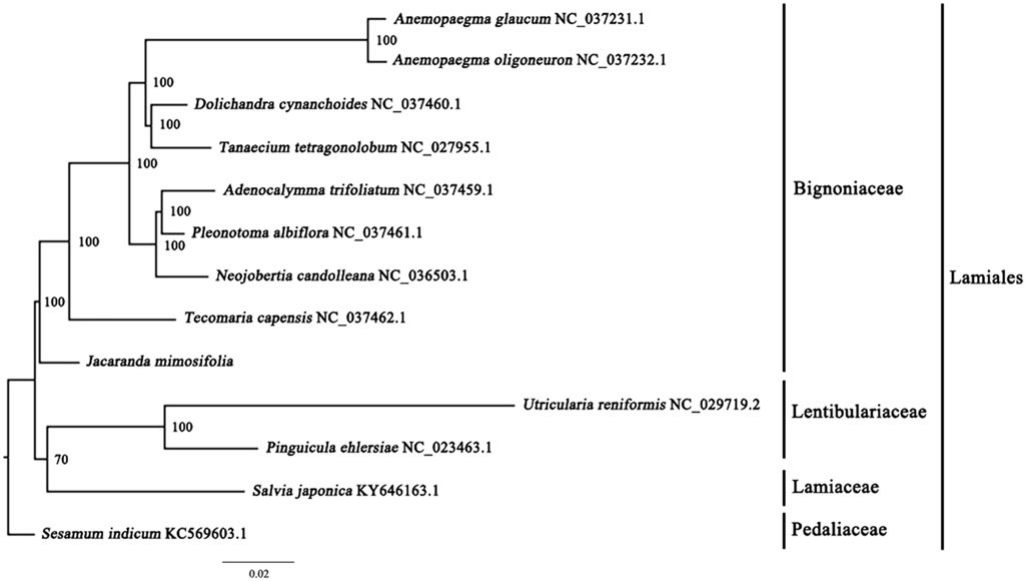
The best ML phylogeny recovered from 13 complete plastome sequences by RAxML. Accession numbers: *Jacaranda mimosifolia* (this study, GenBank accession number: MN450152), *Adenocalymma trifoliatum* NC_037459.1, *Anemopaegma glaucum* NC_037231.1, *Anemopaegma oligoneuron* NC_037232.1, *Dolichandra cynanchoides* NC_037460.1, *Neojobertia candolleana* NC_036503.1, *Pleonotoma albiflora* NC_037461.1, *Tanaecium tetragonolobum* NC_027955.1, *Tecomaria capensis* NC_037462.1; Outgroups: *Pinguicula ehlersiae* NC_023463.1, *Utricularia reniformis* NC_029719.2, *Salvia japonica* KY646163.1, and *Sesamum indicum* KC569603.1.
